# Potential effect of age of BCG vaccination on global paediatric tuberculosis mortality: a modelling study

**DOI:** 10.1016/S2214-109X(19)30444-9

**Published:** 2019-11-07

**Authors:** Partho Roy, Johan Vekemans, Andrew Clark, Colin Sanderson, Rebecca C Harris, Richard G White

**Affiliations:** a TB Modelling Group, TB Centre and Centre for the Mathematical Modelling of Infectious Diseases, Faculty of Epidemiology and Population Health, London School of Hygiene & Tropical Medicine, London, UK; b Faculty of Public Health and Policy, London School of Hygiene & Tropical Medicine, London, UK; c Initiative for Vaccine Research, Immunization, Vaccines and Biologicals, WHO, Geneva, Switzerland

## Abstract

**Background:**

BCG has been recommended at birth in countries with a high tuberculosis burden for decades, yet delayed vaccination is widespread. To support a WHO guidance review, we estimated the potential global tuberculosis mortality benefit of administering BCG on time and consequences of later administration.

**Methods:**

We estimated age-specific BCG coverage in 152 high-burden countries using data from large, nationally representative household surveys, to parameterise a static mathematical model, calibrated to global childhood tuberculosis deaths in 2016. 12 hypothetical scenarios explored the effect of BCG delivery at birth, 6 weeks, 6 months, or 9–12 months, on tuberculosis deaths per global birth cohort by age 15 years, including delivery at the time of the first diphtheria–tetanus–pertussis vaccine (DTP1) or the first measles-containing vaccine (MCV1). We assumed constant vaccine efficacy by age, but varied coverage and degree of vaccination delay, including no delay.

**Findings:**

In 152 high-burden countries, we estimated that BCG coverage in 2016 was 37% at 1 week of age, 67% at 6 weeks, and 92% at 3 years. Modelled scenarios in which 92% BCG coverage was achieved at birth reduced tuberculosis deaths in the global birth cohort by 5449 (95% uncertainty range 218–15 071) or 2·8% (0·1–7·0) by age 15 years. 100% coverage at birth reduced tuberculosis deaths by 16·5% (0·7–41·9). Later administration increased tuberculosis deaths—eg, BCG vaccination at 6 weeks, the recommended age of DTP1, increased tuberculosis deaths by 0·2% (0–0·4), even if BCG reached DTP1 coverage levels (94% at 3 years).

**Interpretation:**

Reducing delays and increasing coverage at birth would substantially reduce global paediatric tuberculosis mortality. Modelled scenarios whereby BCG was administered later in the infant schedule were all estimated to increase tuberculosis deaths, even with increased coverage. The WHO recommendation for BCG at birth should be maintained and emphasised.

**Funding:**

WHO.

## Introduction

BCG vaccine was introduced into WHO's Expanded Programme on Immunization in 1974 and it remains the only tuberculosis vaccine on the market today. It is given to approximately 100 million children worldwide each year.^1^

BCG prevents the most severe forms of childhood tuberculosis, reducing the risk of meningeal and miliary tuberculosis by 85% (95% CI 69–92),^2^ and tuberculosis-associated death by 66% (8–88).^3^ At between US$2–3 per dose, it is a highly cost-effective intervention, estimated to prevent one case of tuberculosis meningitis in the first 5 years of life for every 3435 vaccinations given, and one case of miliary tuberculosis for every 9314 vaccinations given.^1^ Modelling suggests that global BCG shortfalls that occurred in 2015 might lead to an additional 7433 tuberculosis deaths in the affected birth cohort in the first 15 years of life.^4^ Emerging evidence is also suggestive of BCG providing non-specific beneficial effects on all-cause mortality.^5^

BCG has been recommended at birth in countries with a high tuberculosis burden for decades. Currently, 152 low-income and middle-income countries (LMICs) have a policy of universal neonatal vaccination at birth or in the first week of life.^6^ Even so, childhood vaccinations, including BCG, are often delayed until well after the recommended age.^7^ In support of a WHO guidance review and to inform an update to its BCG position paper,^8^ we investigated the potential global tuberculosis mortality benefit of administering BCG on time and modelled the consequences of BCG administered later in the infant schedule.

## Methods

### Mathematical model

A static mathematical model was developed using Microsoft Excel 2016 (Microsoft Corporation, Redmond, WA, USA) to calculate the change in number of tuberculosis deaths per birth cohort in the first 15 years of life (Δ*pTBD*), in each scenario of different age at BCG vaccination, compared with the baseline scenario (scenario A: BCG vaccination coverage by age distribution for the 2016 birth cohort [92% final coverage]):

(equation 1) $$\Delta pTBD=\frac{pTBD_{scenario}}{pTBD_{baseline}}$$

**Research in context**

**Evidence before this study**

WHO estimated that approximately 253 000 children younger than 15 years died from tuberculosis in 2016, including 52 000 deaths in children with HIV. Modelling estimates suggest that the majority of childhood tuberculosis deaths occurred in children younger than 5 years, although the number of these deaths occurring in children younger than 2 years—who are at greatest risk of tuberculosis death—is not known. BCG vaccine has been recommended at birth in high tuberculosis burden countries for decades. However, delays in vaccination are widespread and many vaccines, including BCG, are delivered after the recommended age. Meta-analyses have shown that neonatal BCG vaccination provides protection against meningeal tuberculosis, miliary tuberculosis, and against tuberculosis death. Evidence on whether BCG vaccine efficacy varies by age of administration is extremely scarce. To explore the existing literature on the population-level effect of changes in timing of BCG vaccination on childhood mortality, we searched MEDLINE, Embase, and PubMed for all English language studies published between database inception and June 30, 2016, using MeSH search terms for BCG, children, tuberculosis, and timing. One study estimated the population-level effect of BCG on morbidity, but no studies were identified exploring the population-level effects of changes in the timing of BCG vaccination on tuberculosis mortality.

**Added value of this study**

This study is the first to explore the potential effect of age of BCG vaccination on global paediatric tuberculosis mortality. This study provides evidence of the benefits of administering BCG vaccination at birth, and the potential reduction in global tuberculosis deaths that could be achieved with on-time vaccination. Prospective evaluation of the effect of age of BCG on tuberculosis mortality would be challenging for many reasons and mathematical modelling studies such as this study can provide valuable information, alongside available data, to support vaccine recommendations.

**Implications of all the available evidence**

The recommendation for the birth dose of BCG should be maintained and emphasised. This recommendation is supported by previous literature showing high protection against pulmonary, miliary, and meningeal tuberculosis if given to neonates, and by our evaluation showing that current delays in BCG vaccination are associated with increased global paediatric tuberculosis mortality. Through eliminating these delays and increasing BCG coverage at birth, substantial reductions in global paediatric tuberculosis mortality could be achieved. Our analysis supports the updated WHO position paper on BCG vaccines.

The number of paediatric tuberculosis deaths per birth cohort in the first 15 years of life (*pTBD*) for the baseline and each scenario was estimated by:

(equation 2) $$pTBD=\left(\sum_{\text{t}=0}^{\text{t}=261}{\text{n}}_{\text{t}}\times {\text{R}}_{0-4}\right)+\left(\sum_{\text{t}=262}^{\text{t}=782}{\text{n}}_{\text{t}}\times {\text{R}}_{5-14}\right)$$

Where *t* represents age (weeks), *n*_t_ the number of unprotected children at age *t*, and *R*_0–4_ and *R*_5–14_ were the weekly individual risk of tuberculosis death in unprotected children aged 0–4 years or 5–14 years, respectively (as calculated in equation 7).

In each scenario, *n*_t_ was estimated to be the number of unvaccinated children at age *t* plus the number of children vaccinated but with insufficient immune response to prevent tuberculosis death at age *t:*

(equation 3) $$n_{t}=U_{t}+\left(V_{t}\times \left[1-VE\right]\right)$$

where *U*_t_ is the number of unvaccinated children at age *t, V*_t_ the number of vaccinated children at age *t*, and *VE* vaccine efficacy (proportion), calculated as:

(equation 4) $$U_{t}=\left(\left[BC-D_{t}\right]\times \left[1-Cov_{t}\right]\right)$$

(equation 5) $$V_{t}=\left(BC-D_{t}\right)\times Cov_{t}$$

(equation 6) VE=1-RR

where *BC* is the global annual number of BCG eligible births, *D*_t_ the global number of all-cause deaths in BC at age *t, Cov*_t_ the proportion of BC vaccinated at age *t*, and *RR* the rate ratio of tuberculosis deaths in BCG vaccinated versus unvaccinated neonates.

In the baseline scenario (scenario A; table 1, figure), the model was calibrated to the 2016 WHO estimate of paediatric tuberculosis deaths. In this baseline scenario, the WHO estimate of paediatric tuberculosis deaths was randomly sampled 100 000 times, and used in conjunction with estimates of age-specific BCG vaccine coverage data, the sampled proportion of tuberculosis deaths estimated to occur in children aged 0–4 years and 5–14 years, and other sampled parameters (table 2), to inform the value of the age-specific weekly individual risk of tuberculosis death in unprotected children aged 0–4 years and 5–14 years for each sampled parameter set:

**Table 1:** BCG vaccination scenarios

	**Age of BCG delivery**	**Scenario description**
A	Birth	Baseline scenario, BCG vaccination coverage by age distribution for the 2016 birth cohort
B	Birth	92% immediate coverage at birth
C	Birth	100% immediate coverage at birth
D	6 weeks	0% coverage until 6 weeks of age, then 92% immediate coverage at 6 weeks
E	6 weeks	0% coverage until 6 weeks of age, then 92% final coverage delaying the baseline BCG coverage distribution by 6 weeks
F1	6 weeks	BCG global delivery at the time of DTP1, using DTP1 coverage by age distribution (capped when coverage reached 92%)
F2	6 weeks	BCG global delivery at the time of DTP1, using DTP1 coverage by age distribution (final coverage of 94%)
G	6 months	0% coverage until 6 months of age, then 92% immediate coverage at 6 months
H	6 months	0% coverage until 6 months of age, then 92% final coverage delaying the baseline BCG coverage distribution by 6 months
I1	9 or 12 months	BCG global delivery at the time of MCV1, using MCV1 coverage by age distribution (capped when coverage reached 92%)
I2	9 or 12 months	BCG global delivery at the time of MCV1, using MCV1 coverage by age distribution (final coverage of 93%)
J	12 months	0% coverage until 12 months of age, then 92% immediate coverage at 12 months
K	12 months	0% coverage until 12 months of age, then 92% final coverage delaying baseline BCG coverage distribution by 12 months

Scenario A (baseline scenario) is the BCG vaccination coverage by age distribution for the 2016 birth cohort. Scenarios B–K are hypothetical BCG vaccination scenarios with vaccine delivery from birth to 12 months of age. DTP1=first diphtheria–tetanus–pertussis vaccine dose. MCV1=first measles-containing vaccine dose.

**Figure:**
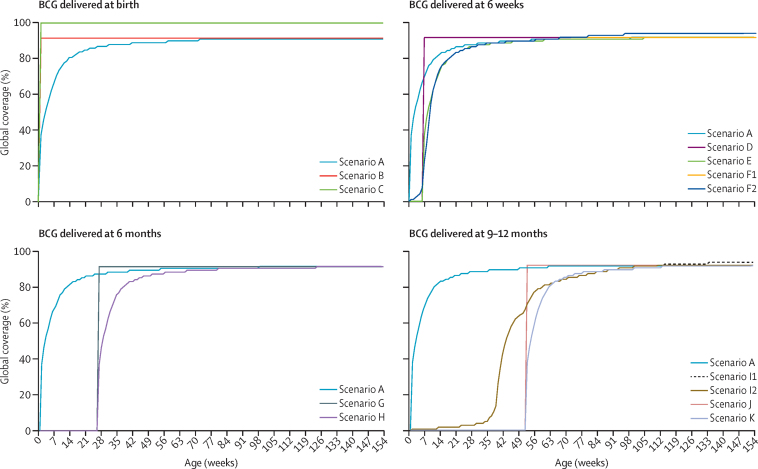
BCG vaccination scenarios Scenario A (baseline scenario) is the BCG vaccination coverage by age distribution for the 2016 birth cohort. Scenarios B–K are hypothetical BCG vaccination scenarios with vaccine delivery from birth to 12 months of age.

**Table 2:** Data inputs and model assumptions

	**Point estimate (95% CI)**	**Source**
**Population characteristics**		
Annual global BCG eligible birth cohort	129·1 million	9
Annual all-cause childhood deaths in birth cohort in children aged 0–4 years	6·8 million	11
Annual all-cause childhood deaths in birth cohort in children aged 5–9 years	643 000	11
Annual all-cause childhood deaths in birth cohort in children aged 10–14 years	560 000	11
HIV-negative paediatric male tuberculosis deaths in 2016	110 000 (75 000–151 000)	13
HIV-negative paediatric female tuberculosis deaths in 2016	91 000 (60 000–129 000)	13
Tuberculosis deaths in children younger than 5 years who had received tuberculosis treatment in 2015 (HIV negative)	2690 (1850–4180)	14
Tuberculosis deaths in children younger than 5 years who had not received tuberculosis treatment in 2015 (HIV negative)	161 000 (108 000–223 000)	14
Tuberculosis deaths in children aged 5–15 years who had received tuberculosis treatment in 2015 (HIV negative)	2050 (1510–3100)	14
Tuberculosis deaths in children aged 5–15 years who had not received tuberculosis treatment in 2015 (HIV negative)	31 500 (18 600–51 400)	14
**Vaccine characteristics**		
Vaccine efficacy against tuberculosis death, %	66% (8–88)	3
Vaccine efficacy by age at administration	Constant regardless of age of administration	Assumption
Duration of protection	15 years	21
Waning of protection	None	Assumption

(equation 7) $$R_{a}=\frac{Mort_{a}}{\Sigma n_{a}}$$

where *a* is the age group (0–4 years or 5–14 years), *Mort*_a_ the number of tuberculosis deaths in each age group, and *∑n*_a_ the number of unprotected person-weeks in each age group.

The calculated age-specific risk of tuberculosis death in unprotected children (*R*_a_) from the baseline scenario was used to estimate the change in number of tuberculosis deaths per birth cohort in the first 15 years of life for each vaccination scenario.

### Data and model assumptions

Data inputs, model assumptions, and sources are summarised in table 2. The global number of BCG eligible births was estimated at 129·1 million, calculated from the annual number of births from 152 LMICs with a policy of universal neonatal vaccination^6^ using UN Population Division (UNPD) birth estimates (2017 revision).^9^ The birth cohort was not adjusted for children born with HIV for whom BCG might be contraindicated, because data on this parameter are limited and likely to be small (with a maximum potential adjustment of <0·2% of birth cohort).^10^ The weekly number of childhood all-cause deaths in the birth cohort was estimated using UNPD mortality estimates for children aged 0–4 years, 5–9 years, and 10–14 years, assuming all-cause deaths were distributed equally by week within each of these age categories.^11^

WHO estimates of the number of global paediatric tuberculosis deaths are summarised in table 2. Given that more than 90% of paediatric HIV infections have been attributed to mother-to-child-transmission,^12^ and that BCG is contraindicated in HIV-infected children unless immunologically stable and on antiretroviral therapy,^8^ we assumed that HIV-positive tuberculosis deaths were not preventable through BCG vaccination. Therefore, only HIV-negative tuberculosis deaths, constituting 80% of all tuberculosis deaths among children aged 0–14 years,^13^ were included in the model.

As per previous modelling studies,^4^ the annual global tuberculosis mortality in children aged 0–14 years was assumed to be equivalent to the tuberculosis mortality of a single global birth cohort over the first 15 years of life. The number of tuberculosis deaths that occurred in the birth cohort between ages 0–4 years and 5–14 years was calculated by applying the proportion of tuberculosis deaths that was estimated to occur in these two age groups in research by Dodd and colleagues^14^ to the 2016 WHO tuberculosis mortality estimate of children aged 0–14 years.^13^

To account for realistic timing of vaccination, we estimated the 2016 country-specific coverage by age of BCG, the first dose of diphtheria–tetanus–pertussis vaccine (DTP1), and the first dose of measles-containing vaccine (MCV1) for 152 LMICs with a policy of universal neonatal BCG vaccination using data from large nationally representative household surveys and previously described methods.^7^ Data from 47 US Agency for International Development Demographic and Health Surveys and 42 UNICEF Multiple Indicator Cluster Surveys were used. Surveys were done between 2005 and 2016, with over 80% of surveys done from 2010 onwards. We assumed no change in vaccine timing between the year of the survey and 2016. For 63 countries without surveys, the mean vaccine timing estimates from country surveys from the same WHO region and WHO mortality stratum^15^ were used. For surveys done before 2016, to scale the study coverage to 2016 coverage values, the vaccine coverage by age distributions were scaled so that the 12-month coverage levels matched the 2016 coverage estimates as reported by WHO/UNICEF Estimates of National Immunization Coverage.^16^ With this approach, the cumulative proportion of the 2016 birth cohort that received each vaccine by week of age from birth until age 3 years was estimated.

12 hypothetical scenarios for timing of BCG vaccination were explored (table 1, figure), including various coverage levels with on-time or delayed vaccination assuming vaccine delivery at birth, 6 weeks, 6 months, or 9–12 months of age, including administration at the time of DTP1 or MCV1.

A meta-analysis of five randomised controlled trials estimated the relative risk of tuberculosis death in BCG-vaccinated neonates versus unvaccinated neonates as 0·34 (95% CI 0·12–0·92),^3^ corresponding to a vaccine efficacy of 66% (95% CI 8–88).

We reviewed published literature exploring BCG efficacy by age at vaccination on tuberculosis and all-cause mortality (appendix p4), identifying two clinical trials,^17^, ^18^ two observational studies,^19^, ^20^ and one systematic review.^5^ Together these studies are suggestive of non-specific effects of BCG, reducing mortality by more than would be expected through the effects on tuberculosis alone, and that the beneficial effects of BCG might decrease as age of vaccination increases. However, the systematic review found that the observational studies were at high risk^20^ or very high risk^19^ of bias, and the clinical trials were from low-birthweight infants from a single setting, so the results might not be generalisable to the global birth cohort. Overall, the systematic review found no conclusive evidence of an association between tuberculosis mortality and age of vaccination, and recommended further research in the area of non-specific effects.^5^ Therefore, in our model, BCG vaccine efficacy against tuberculosis death was assumed constant by age of administration, and non-specific effects were not included.

BCG was modelled as providing all-or-nothing protection, with vaccine efficacy as the proportion of vaccinated individuals completely protected against tuberculosis death. Infant BCG has a protective effect for at least 10 years following vaccination and evidence suggests that this effect might last up to 15 years.^21^ On this basis, BCG protection was assumed to last without waning for the 15-year model time period.

Paediatric tuberculosis is minimally infectious because pulmonary disease tends to be of low-bacillary load,^22^ young children rarely produce a forceful enough cough to expel bacilli,^22^ and extrapulmonary disease is more common than in adults.^23^ Therefore, children rarely contribute to transmission, so it was assumed vaccination did not provide indirect effects, and the direct effects of vaccination were captured using a static mathematical model.

### Uncertainty and sensitivity analyses

Oracle Crystal Ball was used to generate 100 000 iterations of the model, simultaneously sampling from each parameter distribution (table 1). We report median values and a 95% uncertainty range (UR) from these iterations. Where underlying parameter distributions were not stated, log-normal distributions were generated from each point estimate and 95% range using the 2·5%, 50%, and 97·5% quantiles in the rriskDistributions package in the programme R, consistent with previous work.^4^ A summary of each parameter can be found in the appendix (p 5).

In the baseline scenario, a correlation coefficient of 1 (perfect correlation) was assumed between the number of tuberculosis deaths in children who had received tuberculosis treatment and those who were untreated, and also between the numbers of tuberculosis deaths in children aged 0–4 years and 5–14 years.^14^ These assumptions were explored in a sensitivity analysis, which assumed no correlation (coefficient=0). A further sensitivity analysis explored the assumption of uniform risk of tuberculosis death in children aged 0–4 years, and considered the hypothetical situation where 100% of deaths in this age group occurred in children aged 0–2 years. In each case the model was re-run an additional 100 000 times.

### Role of the funding source

WHO provided financial support to the London Schoold of Hygiene & Tropical Medicine TB Modelling Group to conduct this work, contributed to the development of the research question, and reviewed manuscript drafts. WHO had no role in data collection, data analysis, or data interpretation, and writing of this report. The corresponding author had full access to the data in the study and had final responsibility for the decision to submit for publication.

## Results

In 152 LMICs, we estimate that 37% of the 2016 birth cohort received BCG in the first week of life, and 67% coverage was achieved by 6 weeks of age. BCG coverage was estimated to be 90% at 1 year of age and 92% at 3 years of age (table 3). An estimated 7·7% of the birth cohort received DTP1 before the recommended 6 weeks of age, with 50% coverage achieved between 9 and 10 weeks of age. Coverage at 1 year was estimated at 89% and at 3 years was estimated at 94%. Coverage of MCV1 reached 50% between 43 and 44 weeks, with 67% coverage and 93% coverage at 1 year and 3 years of age, respectively (table 3).

**Table 3:** Vaccine coverage by age distributions for the 2016 birth cohort across 152 low-income and middle-income countries for BCG, the first dose of diphtheria–tetanus–pertussis vaccine (DTP1), and the first dose of measles-containing vaccine (MCV1)

	**WHO recommended age of first dose**	**Age by which 50% coverage of the 2016 birth cohort is achieved**	**Estimated coverage of the 2016 birth cohort at 1 year of age**	**Estimated coverage of the 2016 birth cohort at 3 years of age**	**Comments**
BCG	At birth or as soon as possible after birth	Between 2 and 3 weeks of age	90%	92%	An estimated 37% of the 2016 birth cohort received BCG in the first week of life
DTP1	6 weeks	Between 9 and 10 weeks of age	89%	94%	An estimated 7·7% of the 2016 birth cohort received DTP1 before 6 weeks of age
MCV1	9 or 12 months	Between 43 and 44 weeks of age	67%	93%	An estimated 9·6% of the 2016 birth cohort received MCV1 before 9 months of age

The modelled number of HIV-negative tuberculosis deaths in the baseline scenario (scenario A) for the 2016 birth cohort over the first 15 years of life was 200 830 (95% UR 141 457–285 169), consistent with WHO estimates.^13^ Achieving 92% BCG coverage at birth (scenario B), rather than at the current 3 years of age, was estimated to reduce tuberculosis deaths by 2·8% (UR 0·1–7·0) or 5449 fewer tuberculosis deaths (UR 218–15 071) per global birth cohort over the first 15 years of life (table 4). 100% BCG coverage at birth (scenario C) would reduce tuberculosis mortality by 16·5% (0·7–41·9), or 32 758 fewer deaths (1292–90 412).

**Table 4:** Estimated tuberculosis mortality per birth cohort in the first 15 years of life according to different BCG vaccination scenarios

	**Scenario description**	**Total number of childhood tuberculosis deaths per birth cohort in the first 15 years of life (95% UR)**	**Change in number of tuberculosis deaths compared with Scenario A (95% UR)**	**Median percentage change in number of tuberculosis deaths compared with scenario A (95% UR)**
A	Baseline BCG vaccination coverage by age distribution (92% final coverage)	200 830 (141 457 to 285 169)	..	..
B	92% immediate coverage at birth	194 857 (136 943 to 277 490)	−5499 (−15 071 to −218)	−2·8% (−7·0 to −0·1)
C	100% immediate coverage at birth	165 174 (101 880 to 249 423)	−32 758 (−90 412 to −1292)	−16·5% (−41·9 to −0·7)
D	0% coverage until 6 weeks of age, then 92% immediate coverage at 6 weeks	201 103 (141 645 to 285 642)	264 (10 to 724)	0·1% (0 to 0·3)
E	0% coverage until 6 weeks of age, then 92% final coverage delaying the baseline BCG coverage distribution by 6 weeks	207 013 (145 717 to 294 531)	5757 (228 to 15 775)	2·9% (0·1 to 7·3)
F1	BCG global delivery at the time of DTP1, using DTP1 coverage by age distribution (capped when coverage reached 92%)	206 805 (145 590 to 294 274)	5556 (220 to 15 224)	2·8% (0·1 to 7·1)
F2	BCG global delivery at the time of DTP1, using 2015 DTP1 coverage by age distribution (final coverage of 94%)	201 165 (141 703 to 285 809)	332 (11 to 964)	0·2% (0 to 0·4)
G	0% coverage until 6 months of age then 92% immediate coverage at 6 months	221 584 (153 809 to 319 965)	19 426 (769 to 53 226)	9·8% (0·4 to 24·7)
H	0% coverage until 6 months of age, then 92% final coverage delaying the baseline BCG coverage distribution by 6 months	227 281 (156 543 to 331 633)	24 896 (986 to 68 213)	12·5% (0·5 to 31·6)
I1	BCG global delivery at the time of MCV1, using 2015 MCV1 coverage by age distribution (capped when coverage reached 92%)	242 124 (162 805 to 365 403)	39 553 (1567 to 108 364)	19·9% (0·8 to 50·2)
I2	BCG global delivery at the time of MCV1, using 2015 MCV1 coverage by age distribution (final coverage of 93%)	239 091 (161 666 to 358 146)	36 506 (1445 to 99 882)	18·4% (0·7 to 46·3)
J	0% coverage until 12 months of age, then 92% immediate coverage at 12 months	246 749 (164 570 to 376 504)	44 219 (1751 to 121 142)	22·2% (0·9 to 56·1)
K	0% coverage until 12 months of age, then 92% final coverage delaying baseline BCG coverage distribution by 12 months	252 217 (166 424 to 389 637)	49 660 (967 to 136 043)	25·0% (1·0 to 63·1)

UR=uncertainty range. DTP1=first diphtheria–tetanus–pertussis vaccine dose. MCV1=first measles-containing vaccine dose.

In contrast, in all the other modelled scenarios, later BCG vaccination was associated with increased tuberculosis mortality, even if it was associated with improved BCG coverage (table 4). If BCG was globally administered at around 6 weeks of age, it was estimated that 92% instantaneous coverage (scenario D) would result in increased mortality of 0·1% (0–0·3), compared with the current situation, while delaying the baseline distribution of age at BCG by 6 weeks (scenario E) would increase mortality by 2·9% (0·1–7·3). If BCG was delivered globally at the time of DTP1, tuberculosis mortality would increase by 2·8% (0·1–7·1), equivalent to 5556 more deaths (220–15 224) if BCG coverage remained at 92% (scenario F1), or by 0·2% (0–0·4), equivalent to 332 more deaths (11–964) if BCG coverage increased to 94% to match DTP1 coverage (scenario F2). Achieving 92% instantaneous coverage at 6 months (scenario G) was estimated to increase tuberculosis mortality by 9·8% (0·4–24·7), while shifting the baseline distribution of age at BCG by 6 months (scenario H) was estimated to result in an increase of 12·5% (0·5–31·6; table 4). If BCG was to be administered at the time of MCV1 at 9–12 months of age, mortality would increase by an estimated 19·9% (0·8–50·2) if BCG coverage remained at 92% (scenario I1), or by 18·4% (0·7–46·3) if BCG coverage increased to match MCV1 coverage of 93% (scenario I2). Instantaneous 92% coverage at 12 months (scenario J) would increase mortality by an estimated 22·2% (0·9–56·1), while delaying the current baseline distribution by 12 months (scenario K) was estimated to increase mortality by 25·0% (1·0–63·1).

Exploring the assumptions of correlation between number of tuberculosis deaths in children aged 0–4 years and 5–14 years, and correlation between number of treated versus untreated deaths, had a negligible effect on mortality estimates (appendix p 6). When all tuberculosis deaths in children younger than 5 years were modelled to occur between the ages of 0–2 years, 100% BCG vaccination at birth was estimated to reduce tuberculosis deaths by 19·6% (0·8–47·2), while all scenarios of later vaccination resulted in even greater increases in tuberculosis deaths compared with the main analysis (appendix p 6).

## Discussion

In 152 LMICs with a policy of neonatal BCG vaccination, we estimated that 37%, 67%, and 90% of the 2016 birth cohort received BCG by 1 week, 6 weeks, and 1 year of age, respectively. Coverage at 3 years of age was estimated to be 92%. By eliminating delays in BCG administration and achieving 92% BCG coverage at birth, rather than as currently at 3 years of age, we estimated a reduction in tuberculosis deaths in the global birth cohort over the first 15 years of life of 5449 deaths (UR 218–15 071), equivalent to 2·8% (UR 0·1–7·0). Furthermore, achieving 100% BCG coverage at birth, in line with WHO recommendations, would reduce tuberculosis deaths by 32 758 (1292–90 412) or 16% (0·7–42). Later administration would increase tuberculosis deaths in all the modelled scenarios. For example, global administration of BCG at the time of DTP1, recommended at 6 weeks, would increase tuberculosis deaths by 0·2% (0–0·4) even if BCG coverage reached DTP1 coverage levels (94% by age 3 years). The longstanding WHO recommendation for BCG at birth has, therefore, likely averted a substantial number of childhood tuberculosis deaths that would have occurred had BCG been recommended later in the vaccination schedule. Our analysis supports the updated WHO position paper on BCG vaccines, which recommends that in countries with a high incidence of tuberculosis, BCG should be given to all healthy neonates at birth or at the earliest opportunity thereafter.^8^

Childhood tuberculosis remains a substantial global public health problem, with almost 250 000 deaths and more than 1 million new cases in 2017.^24^ The international tuberculosis community has endorsed an ambitious goal of zero childhood tuberculosis deaths.^25^ If this target is to be achieved, vaccination will undoubtedly play a crucial role. Although 100% BCG coverage at birth is unlikely to be achieved, our analyses have shown the importance of both eliminating delays and increasing coverage, and the reductions in childhood tuberculosis mortality that could be achieved in ideal circumstances. Although new tuberculosis vaccines are in development, BCG remains the only tuberculosis vaccine currently available on the global market, and until new vaccines are widely available, efforts to improve the timing and coverage of BCG should be emphasised.

There are several reasons why BCG delivery might be delayed. Children born at home might have infrequent contact with health workers, many countries operate a policy of delayed vaccination for low-birthweight children, and some countries have a policy of not opening multidose BCG vials until a sufficient number of children are present to minimise vaccine wastage (restricted vial opening policies).^26^ In Guinea-Bissau, restricted vial opening policies have resulted in mothers being asked to return on another occasion for their children to receive the vaccine, thereby leading to further delays.^27^ In this setting, when BCG was provided regardless of the number of children present, delays were substantially reduced.^27^ Where clinics vaccinate seven or fewer children daily, production of BCG in 10-dose vials has been estimated to be cost-effective.^28^ Therefore, both policy recommendation and reduced vial size could help encourage vial opening, potentially leading to reduced delays and, therefore, preventable childhood mortality.

Although our research has focused on the reduction of tuberculosis mortality, there is an additional potential reduction in all-cause mortality from administering BCG at birth. However, because the evidence base for non-specific effects of BCG is still emerging and currently inconclusive,^5^ we have not incorporated this outcome into our model. For example, studies reporting non-specific effects have sometimes produced seemingly conflicting results. Although it has been reported that simultaneous administration of BCG and DTP1 at 6 weeks of age might be associated with reduced all-cause mortality,^29^ it has also been reported that BCG at birth reduces all-cause mortality compared with delayed vaccination.^17^ The potential additional effect of BCG on all-cause mortality is an interesting research question, and could be incorporated into future models once improved data from ongoing research become available.

This study has several strengths. It is the first study to explore the potential effect of age of BCG vaccination on global paediatric tuberculosis mortality. The scenarios exploring delivery of BCG vaccination at birth provide evidence of the benefits of on-time vaccination and a theoretical benchmark for the potential reduction in tuberculosis deaths. Secondly, the accuracy of our model estimates was strengthened by use of age-specific vaccine coverage data based on household surveys, rather than using country-level final coverage estimates, which do not take vaccine delays into account. As a result, theoretical scenarios whereby global administration of BCG would be deferred, to coincide with DTP1 or MCV1, account for the fact that vaccine administration is often distributed around, and mainly after, the recommended age, therefore providing more realistic estimates than simply assuming that vaccination takes place only at the recommended age. Finally, methods for estimating the burden of childhood tuberculosis at a global and individual country level are continuously evolving. Although this model was developed using 2016 data, it is set up so that any new global estimates of tuberculosis mortality could be incorporated to produce updated estimates or could be adapted to a specific country with available data.

This study has several limitations. First, we have used the best available estimate of age-specific tuberculosis mortality,^14^ but data to inform this parameter were limited to 0–4 years and 5–14 years age groups. Children younger than 2 years are most at risk of tuberculosis death;^8^ therefore by assuming uniform risk between ages 0–4 years, our model might have underestimated the true mortality benefit of delivering BCG on time at birth, and the consequences of delivering BCG later in the infant schedule. Although a sensitivity analysis modelled a scenario in which all tuberculosis deaths in children younger than 5 years occurred between the ages of 0 and 2 years, more precise data on how tuberculosis deaths are stratified by age could improve future model estimates.

Second, data on BCG vaccine efficacy were limited. The parameter of vaccine efficacy against tuberculosis death^3^ had large confidence intervals, and so the URs presented around each modelled estimate are wide. However, these ranges show the values that might be achieved at different levels of true vaccine efficacy. Therefore, overlapping URs between scenarios should not be interpreted as a lack of difference between scenarios, because regardless of vaccine efficacy, the relative difference between scenarios would remain consistent. Improved estimates of vaccine efficacy against tuberculosis death would result in narrower URs. Additionally, due to absence of conclusive evidence, our model assumed constant vaccine efficacy against tuberculosis death regardless of age of administration. This assumption might not be true because evidence suggests that BCG is more efficacious in individuals not previously sensitised to *Mycobacterium tuberculosis*,^2^ and that potential non-specific effects might decrease with age.^17^, ^19^ However, declining BCG vaccine efficacy with age would be anticipated to increase estimates of tuberculosis mortality with later BCG administration, and would further strengthen the case for the birth dose.

Third, by assuming that tuberculosis deaths in HIV-infected infants are not vaccine-preventable, we have not included the potential effect of BCG vaccination on tuberculosis mortality in this cohort of individuals. BCG is contraindicated in neonates with known HIV infection, unless on antiviral treatment and immunologically stable, because of the increased risk of disseminated BCG disease. Yet, HIV-infected neonates might receive BCG if their HIV status is unknown at birth, the risks and benefits of which have not been accounted for in this research. In addition, coverage in the scenario of BCG vaccination alongside DTP1 might have been overestimated, because HIV status is more likely to be known by this stage, although the number of infants affected is likely to be small.

Finally, because paediatric tuberculosis cases are minimally infectious, we used static modelling. As such, our model does not account for any potential further reduction in cases due to decreased disease transmissibility or indirect vaccine effects, although any potential effect is likely to be small.

In future work, this model could be strengthened by improved information on vaccine efficacy by age and more stratified estimates of age-specific tuberculosis mortality. Whether smaller multidose vials or unrestricted vial-opening policies would lead to improved coverage and reduced delays should also be investigated.

In conclusion, BCG remains a cornerstone of childhood tuberculosis prevention policy, but delays in its administration still exist in many settings. Eliminating these delays and increasing coverage at birth would lead to a substantial reduction in global paediatric tuberculosis mortality. Modelled scenarios whereby BCG is administered later in the infant schedule were all estimated to increase tuberculosis deaths. The WHO recommendation for BCG at birth should be maintained and emphasised.
