# Supplementary material for: Potential effect of age of BCG vaccination on global paediatric tuberculosis mortality: a modelling study
Source: Lancet Glob Health. 2019 Nov 7;7(12):e1655–63. doi: 10.1016/S2214-109X(19)30444-9 (PMC7024998; doi:10.1016/S2214-109X(19)30444-9)
Supplement: Supplementary appendix [file mmc1.pdf]

THE LANCET
Global Health

Supplementary appendix

This appendix formed part of the original submission and has been peer reviewed. We post it as supplied by the authors.

Supplement to: Roy P, Vekemans J, Clark A, Sanderson C, Harris RC, White RG. Potential effect of age of BCG vaccination on global paediatric tuberculosis mortality: a modelling study. *Lancet Glob Health* 2019; **7:** e1655–63.

The potential impact of age of BCG vaccination on global paediatric tuberculosis mortality: A modelling study

Web Extra Material

Authors: Partho Roy, Johan Vekemans, Andrew Clark, Colin Sanderson, Rebecca C Harris and Richard G White**

**Joint senior authors*

Table of Contents

Literature review to explore whether BCG vaccine efficacy varies by age of administration: Search strategy & sifting process. **............ *2***

Figure A1: Sifting Process for literature review 3

Table A1: Table of studies included in literature review 4

Summary of model parameters used in uncertainty analysis. **............ *5***

Table A2: Summary of model parameters 5

Sensitivity analyses **............ *6***

Table A3: Results of sensitivity analyses 6

References **............ *7***

Literature review to explore whether BCG vaccine efficacy varies by age of administration: Search strategy & sifting process.

1. (bcg or bcg vaccin or BCG immuni* or bacillus calmette guerin or bacillus calmette-guerin or bacillus calmette guerin vaccin* or bacillus calmette guerin immuni* or bacillus calmette-guerin vaccin* or bacillus calmette-guerin immuni* or tuberculosis vaccin* or tuberculosis immuni* or TB vaccin* or TB immuni*).mp. [mp=ti, ab, ot, nm, hw, kf, px, rx, ui, tn, dm, mf, dv, kw]*

2. exp tuberculosis vaccines/ or exp bcg vaccine/

3. (tuberculosis or tb or miliary tuberculosis or miliary TB or TB mening or mening* TB or mycobacterium tuberculosis).mp. [mp=ti, ab, ot, nm, hw, kf, px, rx, ui, tn, dm, mf, dv, kw]*

4. exp Tuberculosis/ or exp Tuberculosis, Meningeal/ or exp Mycobacterium tuberculosis/ or exp Tuberculosis, Miliary/

5. (p?ediatric or child or infan* or neonat*).mp. [mp=ti, ab, ot, nm, hw, kf, px, rx, ui, tn, dm, mf, dv, kw]*

6. exp Child, Preschool/ or exp Infant, Newborn/ or exp Child/ or exp Adolescent/ or exp Pediatrics/ or exp Infant/

7. (delay or postpon* or catch-up or Catch-up or late or early).mp. [mp=ti, ab, ot, nm, hw, kf, px, rx, ui, tn, dm, mf, dv, kw]*

8. exp time/ or exp time factors/

9. 1 or 2

10. 3 or 4

11. 5 or 6

12. (delay or postpon* or catch-up or Catch-up or late or early or tim*).mp. [mp=ti, ab, ot, nm, hw, kf, px, rx, ui, tn, dm, mf, dv, kw]*

12. 8 or 16

13. 9 and 10 and 11 and 12

14. limit 13 to humans

15. limit 14 to (clinical study or clinical trial, all or clinical trial or comparative study or controlled clinical trial or meta analysis or multicenter study or observational study or pragmatic clinical trial or randomized controlled trial or systematic reviews)

16. remove duplicates from 15

Figure A1: Sifting Process for literature review

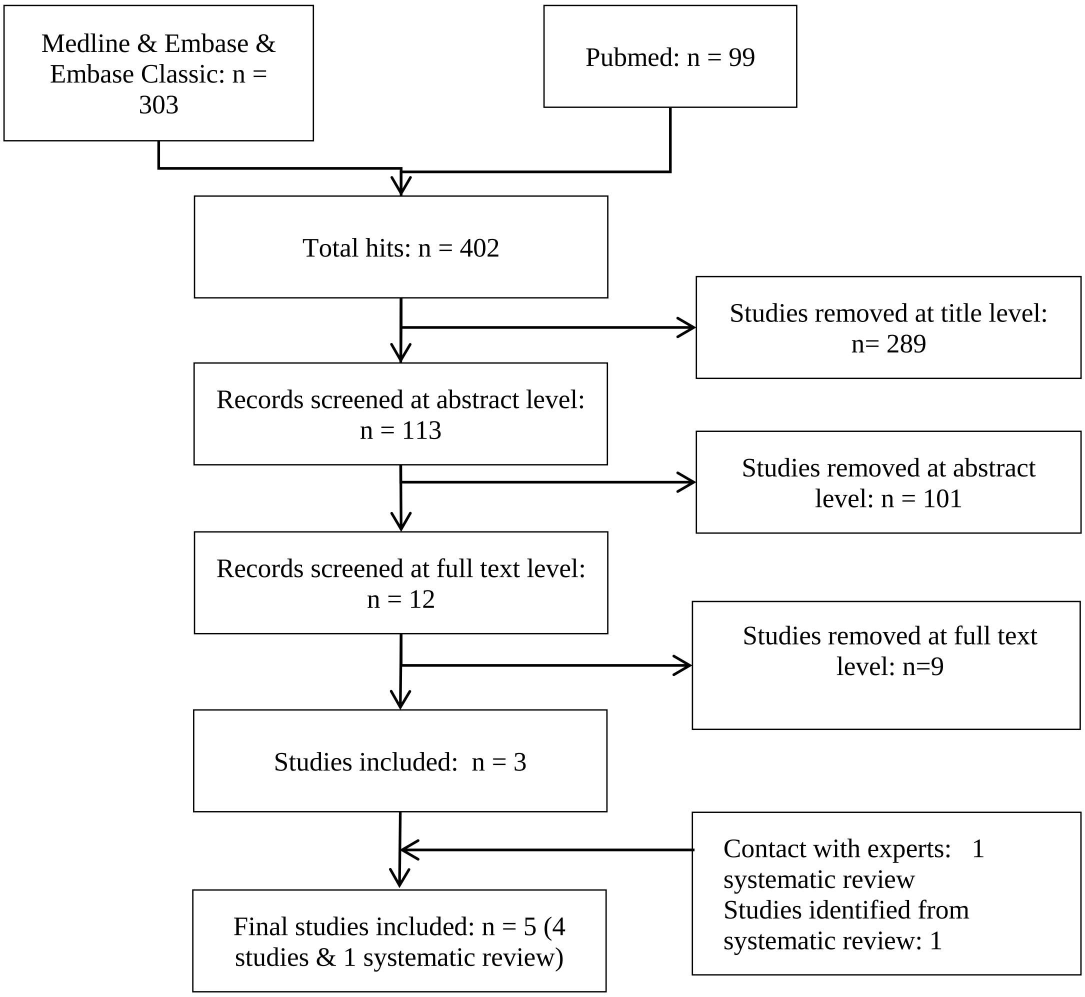

Table A1: Table of studies included in literature review

Country (study years)	Study description	Main outcome/finding/limitation	Reference
Guinea Bissau (2004 – 2008)	RCT comparing BCG at birth vs. delayed BCG in low birth weight (LBW) infants. BCG is routinely delayed for LBW infants in this setting. 2320 infants randomised to receive BCG at birth (intervention group) or delayed BCG (control group).	BCG significantly reduces all-cause mortality at 4 weeks of age (Mortality rate ratio 0.55 [95% CI 0.34 – 0.89]). No significant reduction in mortality at 2, 6 and 12 months of age.<br>Study participants were all LBW infants, therefore questionable whether results are generalizable to normal birth weight infants. Unclear whether infants were premature or at term, therefore limited generalisability.	[1]
Guinea Bissau (2002 – 2004)	This study combines the findings from [1] with n = 105 participants from two trial sites that used exactly the same methodology.	BCG significantly reduces all-cause mortality up to 2 months of age (MRR 0.67 [95% CI 0.47 – 0.95]).<br>Study participants were all LBW infants, therefore questionable whether results are generalizable to normal birth weight infants. Unclear whether infants were premature or at term, therefore limited generalisability.	[2]
Guinea Bissau (1989 – 1999)	Retrospective observational study of community and hospital records of 845 LBW infants, 182 of whom received BCG in first week of life.	BCG given in the first week of life has a greater vaccine efficacy against all-cause mortality than BCG given after first week of life, up to 6 months of age. Vaccine efficacy is 89% (95% CI 50 – 97%) in first week of life, compared to 70% (95% CI 35 – 86%) after first week.<br>Several potential sources of selection bias: Information on birth weight and vaccination status available on less than one third of births in study area. Residual confounding still possible e.g. children with medical problems potentially less likely to receive vaccination.	[3]
Bangladesh (1986 – 2001)	Observational study of Demographic & Health survey data from 70 Bangladeshi villages comprising 36000 children with follow up to 5 years of age.	BCG vaccination does not reduce mortality up to 9 months of age, but is significantly associated with increased mortality between 9 months and 5 years (Hazard ratio (HR) 2.12 [95% CI 1.28 – 3.51]).<br>Children vaccinated between 0 – 60 days, and between 61 – 180 days had significantly lower risk of death compared to unvaccinated (Hazard ratios 0.2, and 0.59 respectively).<br>Children vaccinated between 6 months and 1 year (HR 1.12 (0.68-1.8) and 1 year and 5 years (HR 1.36 (0.86 – 2.14) have increased risk of death compared to unvaccinated, but not significant.<br>Study unable to differentiate between vaccination with BCG and DTP which were co-administered. Confounding variables such as child's health or socio economic status not controlled for.	[4]
n/a	Systematic review regarding non-specific effects of vaccines	"The average age at which BCG vaccination was administered varied across studies. However, there is not strong evidence of a pattern of association between observed effects on mortality and age of vaccination."	[5]

Summary of model parameters used in uncertainty analysis.

Table A2: Summary of model parameters

**Parameter**	**Data mid-point (range)**	**Median value sampled from distribution (95% range)**
Rate ratio of TB deaths in vaccinated vs. unvaccinated neonates	0.34<br>(0.12 to 0.92)	0.34<br>(0.12 to 0.94)
HIV negative TB deaths, males, aged 0 – 14 years	110 000<br>(75 000 to 151 000)	109 970<br>(78 534 to 153 832)
HIV negative TB deaths, females, aged 0 – 14 years	91 000<br>(60 000 to 129 000)	90 888<br>(62 802 to 131 649)
Number of TB deaths in children aged 0 – 4 years, HIV negative, did not receive treatment	161 000<br>(108 000 to 223 000)	161 062<br>(113 930 to 227 391)
Number of TB deaths in children aged 0 – 4 years, HIV negative, received treatment	2 690<br>(1 850 to 4 180)	2 697<br>(1 815 to 4 003)
Number of TB deaths in children aged 5-14 years, HIV negative, did not receive treatment	31 500<br>(18 600 to 51 400)	31 540<br>(19 011 to 52 226)
Number of TB deaths in children aged 5 – 14 years, HIV negative, received treatment	2 050<br>(1 510 to 3 100)	2 054<br>(1 488 to 2 833)

Sensitivity analyses

Main analysis: Number of TB deaths in children under 5 perfectly correlated (coefficient of 1) with children aged 5 – 14, and number of TB deaths in children who had received treatment for TB perfectly correlated (coefficient of 1) with number of children who did not receive treatment for TB.
Sensitivity Analysis 1: No correlation (coefficient of zero) between number of TB deaths in children under 5 and children aged 5 – 14 years.
Sensitivity Analysis 2: No correlation (coefficient of zero) between number of TB deaths in children who had received treatment for TB and children who did not receive treatment for TB
Sensitivity Analysis 3: All TB deaths in children less than 5 years were modelled to occur between the ages of 0-2.

Table A3: Results of sensitivity analyses

Scenario	Scenario Description	Percentage change in number of TB deaths compared to Scenario A (Median, 95% UR)			
		Main Analysis	Sensitivity Analysis 1	Sensitivity Analysis 2	Sensitivity Analysis 3
B	92% immediate coverage at birth.	-2.8 (-7.0 to -0.1)	-2.7 (-7.0 to -0.1)	-2.8 (-7.0 to -0.1)	-6.5 (-15.3 to -0.3)
C	100% immediate coverage at birth.	-16.5 (-41.9 to -0.7)	-16.4 (-41.9 to -0.6)	-16.5 (-42.0 to -0.7)	-19.6 (-47.2 to -0.8)
D	0% coverage until six weeks of age, then 92% immediate coverage at six weeks.	0.1 (0.0 to 0.3)	0.1 (0.0 to 0.3)	0.1 (0.0 to 0.3)	0.4 (0.0 to 0.9)
E	0% coverage until six weeks of age, then 92% final coverage delaying the baseline BCG coverage distribution by six weeks.	2.9 (0.1 to 7.3)	2.9 (0.1 to 7.3)	2.9 (0.1 to 7.3)	6.8 (0.3 to 16.1)
F	BCG global delivery at the time of DTP1, using DTP1 coverage by age distribution (capped when coverage reached 92%).	2.8 (0.1 to 7.1)	2.8 (0.1 to 7.1)	2.8 (0.1 to 7.1)	6.6 (0.3 to 15.8)
F2	BCG global delivery at the time of DTP1, using DTP1 coverage by age distribution (final coverage of 94%).	0.2 (0.0 to 0.4)	0.1 (0.0 to 0.4)	0.2 (0.0 to 0.4)	5.6 (0.2 to 13.0)
G	0% coverage until six months of age then 92% immediate coverage at six months.	9.8 (0.4 to 24.7)	9.7 (0.4 to 24.7)	9.8 (0.4 to 24.7)	23.1 (1.0 to 54.8)
H	0% coverage until six months of age, then 92% final coverage delaying the baseline BCG coverage distribution by six months.	12.5 (0.5 to 31.6)	12.4 (0.5 to 31.6)	12.6 (0.5 to 31.7)	29.4 (1.3 to 69.7)
I	BCG global delivery at the time of MCV1, using MCV1 coverage by age distribution (capped when coverage reached 92%).	19.9 (0.8 to 50.2)	19.7 (0.8 to 50.2)	19.9 (0.8 to 50.3)	46.9 (2.0 to 111.3)
I2	BCG global delivery at the time of MCV1, using MCV1 coverage by age distribution (final coverage of 93%).	18.4 (0.7 to 46.3)	18.2 (0.7 to 46.4)	18.4 (0.7 to 46.4)	46.5 (2.0 to 110.1)
J	0% coverage until 12 months of age, then 92% immediate coverage at 12 months.	22.2 (0.9 to 56.1)	22.1 (0.9 to 56.1)	22.3 (0.9 to 56.3)	52.5 (2.2 to 124.5)
K	0% coverage until 12 months of age, then 92% final coverage delaying baseline BCG coverage distribution by 12 months.	25.0 (1.0 to 63.1)	24.8 (1.0 to 63.1)	25.0 (1.0 to 63.2)	58.4 (2.5 to 138.6)

References

1. Aaby P, Roth A, Ravn H, et al. Randomized trial of BCG vaccination at birth to low-birth-weight children: beneficial nonspecific effects in the neonatal period? *J Infect Dis* 2011; **204**(2): 245-52.
2. Biering-Sorensen S, Aaby P, Napirna BM, et al. Small randomized trial among low-birth-weight children receiving bacillus Calmette-Guerin vaccination at first health center contact. *Pediatr Infect Dis J* 2012; **31**(3): 306-8.
3. Roth A, Jensen H, Garly M-L, et al. Low Birth Weight Infants and Calmette-Guérin Bacillus Vaccination at Birth. *The Pediatric Infectious Disease Journal* 2004; **23**(6): 544-50.
4. Breiman RF, Streatfield PK, Phelan M, Shifa N, Rashid M, Yunus M. Effect of infant immunisation on childhood mortality in rural Bangladesh: analysis of health and demographic surveillance data. *The Lancet* 2004; **364**(9452): 2204-11.
5. Higgins J, Soares-Weiser K, Reingold A. Systematic review of the non-specific effects of BCG, DTP and measles containing vaccines. 2014. https://www.who.int/immunization/sage/meetings/2014/april/3_NSE_Epidemiology_review_Report_to_SAGE_14_Mar_FINAL.pdf?ua=1 (accessed 22 June 2016).
